# Trends in diabetic retinopathy screening attendance and associations with vision impairment attributable to diabetes in a large nationwide cohort

**DOI:** 10.1111/dme.14425

**Published:** 2020-11-01

**Authors:** J. G. Lawrenson, E. Bourmpaki, C. Bunce, I. M. Stratton, P. Gardner, J. Anderson, EROS Study Group

**Affiliations:** ^1^ City, University of London London UK; ^2^ Kings College London London UK; ^3^ Gloucestershire Retinal Research Group Cheltenham UK; ^4^ Public Health England London UK; ^5^ Homerton University Hospital NHS Trust London UK

## Abstract

**Aims:**

To investigate diabetic retinopathy screening attendance and trends in certified vision impairment caused by diabetic eye disease.

**Methods:**

This was a retrospective study of attendance in three urban UK diabetic eye screening programmes in England. A survival analysis was performed to investigate time from diagnosis to first screen by age and sex. Logistic regression analysis of factors influencing screening attendance during a 15‐month reporting period was conducted, as well as analysis of new vision impairment certifications (Certificate of Vision Impairment) in England and Wales from 2009 to 2019.

**Results:**

Of those newly registered in the Routine Digital Screening pathway (*n* = 97 048), 80% attended screening within the first 12 months and 88% by 36 months. Time from registration to first eye screening was longer for people aged 18–34 years, and 20% were unscreened after 3 years. Delay in first screen was associated with increased risk of referable retinopathy. Although 95% of participants (*n* = 291 296) attended during the 15‐month reporting period, uptake varied considerably. Younger age, social deprivation, ethnicity and duration of diabetes were independent predictors of non‐attendance and referable retinopathy. Although the last 10 years has seen an overall reduction in vision impairment certification attributable to diabetic eye disease, the incidence of vision impairment in those aged <35 years was unchanged.

**Conclusions:**

Whilst the majority of participants are screened in a timely manner, there is considerable variation in uptake. Young adults, have sub‐optimal attendance, and levels of vision impairment in this population have not changed over the last 10 years. There is an urgent need to explore barriers to/enablers of attendance in this group to inform policy initiatives and tailored interventions to address this issue.

**What's new?**

- Improvements in diabetes care and the introduction of the National Health Service Diabetic Eye Screening Programme have led to a progressive decline in certified vision impairment caused by diabetic eye disease.
- The annual incidence of new certifications for vision impairment in young adults (age <35 years) has not seen the decline that has occurred in other age groups over the 10‐year reporting period. Although we cannot assume causality we also report sub‐optimal screening uptake in this population.
- There is an urgent need to understand factors influencing retinopathy screening attendance in young adults to inform targeted interventions and policy initiatives to improve uptake.

## 1 INTRODUCTION

The number of people with diabetes in the UK has more than doubled over the past two decades,^1^ with 3.8 million (~6%) of the population currently diagnosed with diabetes. Diabetic eye disease (comprising diabetic retinopathy and diabetic macular oedema) is a microvascular complication of type 1 and type 2 diabetes mellitus. In 2012, a meta‐analysis of 35 population studies estimated the overall prevalence of any retinopathy in those with diabetes to be 35%, with a substantially higher prevalence in those with type 1 diabetes.^2^

Despite the availability of effective treatments, diabetic eye disease remains one of the most common causes of certified blindness in people of working age in the UK and throughout the world.^3^, ^4^ At the time of diagnosis of type 2 diabetes some people already have referable diabetic eye disease. A significant proportion of vision loss is avoidable through early detection and timely treatment. Systematic screening for retinopathy for people with diabetes has long been endorsed as an important public health intervention.^5^ Screening for sight‐threatening retinopathy has been shown to be both clinically effective and cost‐effective.^6^, ^7^

In the UK, diabetic retinopathy screening is administered by the National Screening Committee. In England, the National Diabetic Eye Screening Programme (NDESP) provides screening for all people with diabetes aged ≥12 years through 57 regional Diabetic Eye Screening Programme (DESPs). National programmes in Scotland, Wales and Northern Ireland serve the devolved administrations using a common national service specification. The NDESP uses an electronic system known as the ‘General Practice to Diabetic Retinopathy Screening’ that automatically shares information between general practice databases and the local DESPs. Each screening programme receives a monthly list of those eligible for screening from general practices in their area. All new patients are invited for screening within 3 months of notification. The majority of people are screened through the Routine Digital Screening pathway and are invited for annual screening with digital retinal photography. If sight‐threatening retinopathy is identified, then the person is either monitored more closely in a digital surveillance clinic, or is referred to the Hospital Eye Service for further assessment and possible treatment.

Given the importance of screening attendance for reducing the risk of sight loss amongst people with diabetes, it is essential that DESPs provide consistent and equitable access for the target population. The NDESP defines quality standards for diabetic eye screening^8^ that are monitored through the submission of quarterly and annual reports. The standards state that all newly diagnosed people with diabetes should be offered a screening appointment within 3 months and defines performance thresholds for screening uptake. The minimum acceptable standard is 75% uptake, with 85% considered to be achievable. Although the most recent annual data for England (2018–2019)^9^ reported an overall uptake of 83%, rates vary between programmes (range 74–92%). Previous research has identified a number of modifiable and non‐modifiable risk factors associated with poor screening attendance including younger age, lower socio‐economic status, type of diabetes, ethnicity, lack of awareness of the importance of screening and confusion between diabetic eye screening and routine eye care.^10^, ^11^, ^12^, ^13^, ^14^

Although type 1 is the predominant form of diabetes in young adults, the emerging epidemic of younger adults with type 2 diabetes^15^ presents a particular challenge. Despite improvements in disease management and shorter duration of diabetes, the incidence of sight‐threatening retinopathy is significantly higher in young adults with type 2 diabetes than in an age‐matched control group with type 1 diabetes.^16^, ^17^ The aim of the present study was to investigate the variability in uptake of first screening invitation across age groups within three large urban screening programmes in England and to explore the demographic factors that influence subsequent attendance. We also present a 10‐year retrospective analysis of trends in vision impairment certifications attributable to diabetic eye disease in England and Wales by age.

## 2 PARTICIPANTS AND METHODS

A retrospective analysis of diabetic eye screening uptake was performed from anonymized data extracted from three urban regional screening programmes in England. We also undertook a retrospective analysis of new certifications of sight impairment or severe sight impairment, where diabetic eye disease was the single main cause, for England and Wales, over a 10‐year period from 2009 to 2019.

### 2.1 Screening attendance across age groups

Data were extracted from three screening programmes: 1) North East London; 2) South East London and; 3) Birmingham, Solihull and Black Country. These programmes serve an ethnically diverse population of over 300 000 people with diabetes, and include populations with higher than average levels of social deprivation.

Screening attendance was explored using two different approaches: i) time from registration with the screening programme to first screening attendance (Cohort A); and ii) screening uptake within a 15‐month study period, to capture at least one screening episode during a ‘screening cycle’. Although a screening cycle takes just over 12 months, we extended the reporting period to 15 months to capture those invited at the end of the screening cycle (Cohort B).

Data were extracted from the OptoMize® (Northgate Public Services) screening software platform. For Cohort A, we obtained data on all newly registered participants who were eligible for the Routine Digital Screening pathway from 1 January 2016 until 31 December 2018. Information on screening attendance was collected until 30 April 2019. This allowed a 40‐month reporting period.

For Cohort B, data were collected from all participants who were on register on 1 February 2018 and eligible for the Routine Digital Screening pathway. Screening attendance was recorded for a 15‐month period to 30 April 2019.

The following information was collected for both cohorts: sex; age; self‐reported ethnicity (based on the 2001 UK census standard for classifying the ethnic composition of the communities); type of diabetes; duration of diabetes; retinopathy grading; and Index of Multiple Deprivation (IMD; linked from the participant's postcode by the screening programme prior to providing the data). Based on the retinopathy grading, we defined ‘referable retinopathy’ as a grade requiring closer observation within the DESP through digital surveillance, or referral to an ophthalmologist. This was specified by the most severe retinopathy grade in either eye.

### 2.2 Vision impairment certification

The registration process for certification as sight‐impaired (partially sighted) or severely sight‐impaired (blind) in England and Wales involves the completion of a designated Certificate of Vision Impairment form. The form triggers a referral for a social care assessment if the individual is not yet known to social services. In addition, a copy of the form is sent to the Certifications Office, at Moorfields Eye Hospital, London for anonymized epidemiological research. Certification of vision impairment is dependent on specific visual acuity and field of vision criteria. Further details on the criteria used for certification can be found in a recent publication.^4^ The total number of new certifications for sight impairment and severe sight impairment from 2009/2010 to 2018/2019 for England and Wales were recorded and the number of new certifications that had diabetic eye disease (including retinopathy and maculopathy) as the main cause of certifiable vision impairment were documented. Annual population estimates based on the mid‐year estimates for England and Wales were obtained from the Office of National Statistics and used to calculate certification rates per 100 000 population.

### 2.3 Statistical analysis

Participants’ demographic characteristics were analysed using descriptive statistics. For analysis of first screening attendance Kaplan–Meier plots were used to explore the proportion of people attending in each age strata at registration (12–17, 18–34, 35–59 and ≥60 years) against time from registration. Univariate and multiple fixed‐effects logistic regression analysis was used to investigate the association between screening uptake and explanatory demographic factors including: age, sex, self‐reported ethnicity and socio‐economic deprivation (IMD data were obtained using the postcode). The general practice code was included as a random effect. Similarly, fixed effects logistic regression was used to identify risk factors associated with the detection of referable retinopathy. All data were processed and analysed using stata/ic 15.1 statistical software.

### 2.4 Ethics

Analysis of anonymized data did not require ethical approval, but we obtained approval from each Research and Development Department of the National Health Service (NHS) Trusts providing data. All people in the Certificate of Vision Impairment dataset had provided explicit consent for anonymized data to be sent to the Certifications Office for research purposes.

## 3 RESULTS

### 3.1 Screening attendance across age‐groups

**Figure 1:**
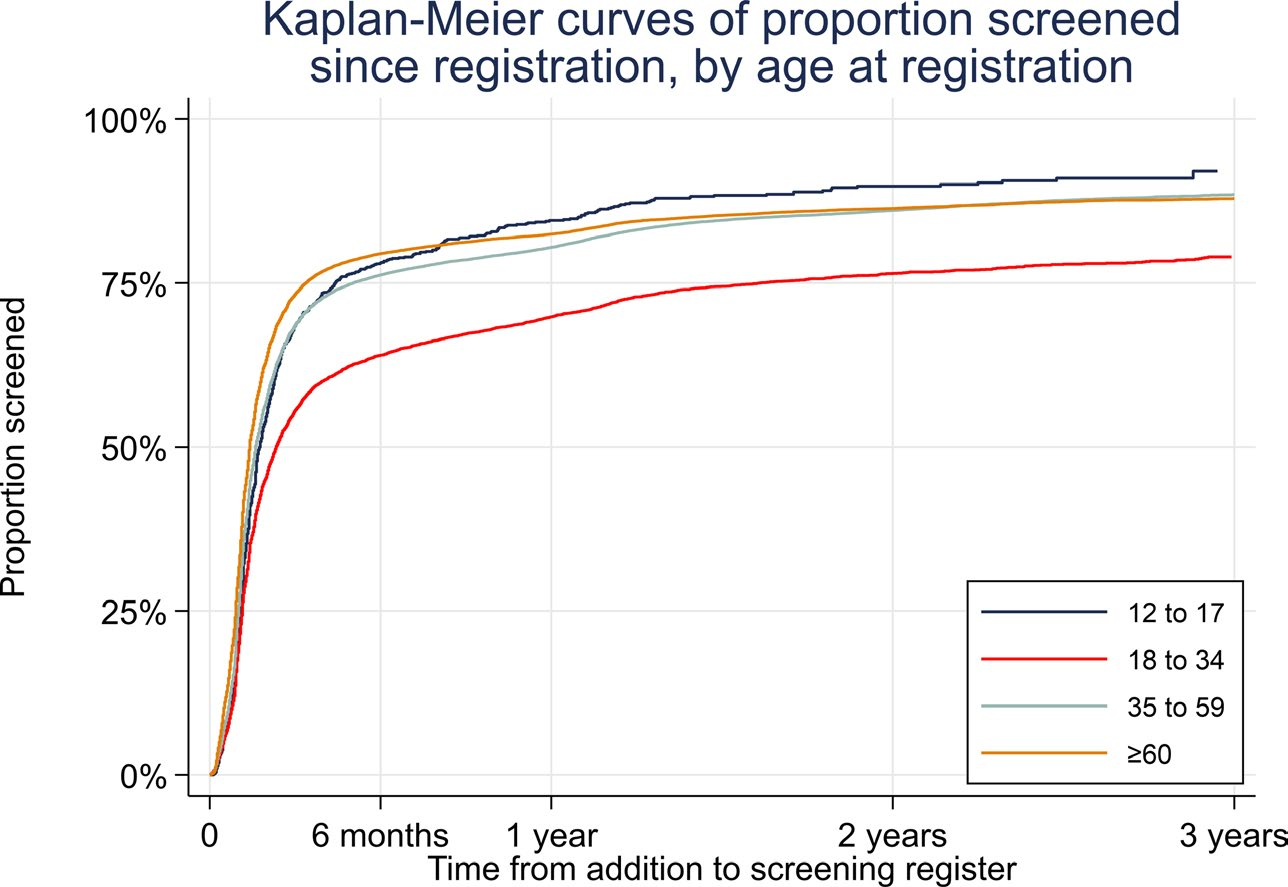
Kaplan–Meier plot of time to first screening event after registration by age strata: 12–17 years, 18–34 years, 35–59 years and ≥60 years (Cohort A).

Age at registration, sex and postcode (for IMD analysis) are mandatory data fields and were available for almost all records. Ethnicity data were missing from 21% of records, type of diabetes for 35% and duration of diabetes for 45%. For Cohort A, data from 97 048 newly registered participants were included in the analysis (Table 1). From this cohort 15 919 (16%) did not attend their first screening event within 40 months. Referable retinopathy was detected in approximately 3% of those screened. For the Kaplan–Meier analysis of time to first screening event, data were available for 1025 people aged 12–17 years, 8710 aged 18–34 years, 50 928 aged 35–59 years and 36 385 aged ≥60 years. Of these, 78% of participants attended their first screening event within 6 months of registration, 80% by year 1 and 88% by 3 years post‐registration (Figure 1). Time to first screening was greater for participants aged 18–34 years at registration and 20% of this group had not attended screening at 3 years. The time to the first screening event was similar for men and women. Logistic regression analysis showed that people aged 18–34 years at registration were more likely to have referable retinopathy than those aged 35–59 years [odds ratio (OR) 1.26, 95% CI 1.10–1.45]. The longer the interval between registration and screening attendance, the more likely the individual was to have referable retinopathy [attendance at 12–35 months compared to attendance within 2 months (OR 1.83, 95% CI 1.57–2.13)].

**Table 1:** Demographic characteristics of newly registered people in the Routine Digital Screening pathway (Cohort A; *N* = 97 048).

Characteristic		Missingness, *n* (%)
Mean (sd) age at registration, years	54.9 (15.4)	‐
Age at registration in groups	*n* (%)	
12–17 years	1025 (1.1)	‐
18–23 years	1269 (1.3)	
24–29 years	2639 (2.7)	
30–35 years	4802 (4.9)	
36–41 years	8460 (8.7)	
42–47 years	12 078 (12)	
48–53 years	15 181 (16)	
54–59 years	15 209 (16)	
≥60 years	36 385 (38)	
Total, *N* = 97 048		
Sex	*n* (%)	
Female	41 422 (45)	4385 (4.5)
Male	51 227 (55)	
Unknown	14 (0.0)	
Total, *N* = 92 663		
Ethnicity	*n* (%)	
White	31 360 (41)	20 446 (21.1)
Mixed	1533 (2.0)	
Asian or Asian British	21 822 (29)	
Black or Black British	13 472 (18)	
Other/not stated/unknown	8415 (11)	
Total, *N* = 76 602		
National IMD decile groups (group 1 most deprived, group 4 least deprived)	*n* (%)	
Group 1: (deciles 1–2)	41 949 (43)	319 (0.3)
Group 2 (deciles 3–4)	28 726 (30)	
Group 3 (deciles 5–6)	13 454 (14)	
Group 4: (deciles 7–10)	12 600 (13)	
Total, *N* = 96 729		
Diabetes type	*n* (%)	
Type 1	3209 (5.1)	34 192 (35.2)
Type 2	57 757 (92)	
Not specified	1742 (2.8)	
Other	148 (0.3)	
Total, *N* = 62 856		
Time from diagnosis of diabetes to registration		
<5 years	48 011 (90)	43 661 (45.0)
5–9 years	2348 (4.4)	
10–19 years	2209 (4.1)	
≥20 years	819 (1.5)	
Total, *N* = 53 387		

Abbreviation: IMD, Index of Multiple Deprivation.

**Figure 2:**
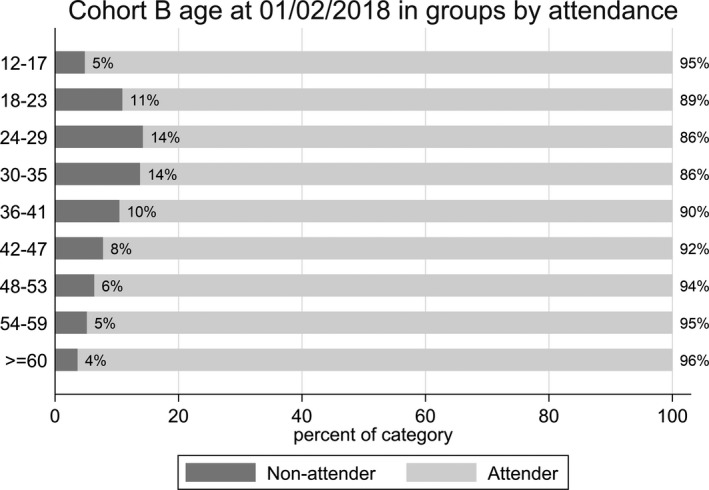
Stacked bar plot of all people eligible for Routine Digital Screening from three Diabetic Eye Screening Programmes, by age group and attendance.

For Cohort B, 291 296 people on screening registers and eligible for the Routine Digital Screening pathway were included in the analysis (Table 2). From this cohort, 14 960 (5%) did not attend for screening during the 15‐month reporting period, with the remaining 276 335 (95%) attending. Data were excluded for one person, who attended but left before screening could be performed. From those who attended a screening event, 93% were recalled for annual screening. Referable retinopathy was detected in 2.6%, who required either closer surveillance within the DESP or referral to ophthalmology. An urgent referral to the Hospital Eye Service was necessary for 0.2%. Ninety percent (260 633) of the cohort attended their first screening appointment within 15 months of registration with the DESP. There was significant variation in uptake among groups stratified by age (Figure 2), with 14% non‐attendance in those aged 24–29 and 30–35 years compared to 4% non‐attendance in those aged >60 years. Uptake was higher among people who had attended their first screening appointment within 15 months of initial registration, with only 3% of people who attended their first screening appointment not attending, compared to 24% who missed their first screening appointment.

**Table 2:** Demographic characteristics of all people in the Routine Digital Screening pathway (Cohort B; *N* = 291 296).

Characteristic		Missingness, *n* (%)
Mean (sd) age at 1 February 2018, years	61.8 (14)	‐
Age 1 February 2018, *n* (%)		
12–17 years	1366 (0.5)	‐
18–23 years	1869 (0.6)	
24–29 years	2923 (1.0)	
30–35 years	5562 (1.9)	
36–41 years	11 690 (4.0)	
42–47 years	21 757 (7.5)	
48–53 years	34 569 (12)	
54–59 years	44 628 (15)	
≥60 years	166 932 (57)	
Total, *N* = 291 296		
Sex		
Female	132 647 (46)	2909 (1.0)
Male	155 717 (54)	
Unknown	23 (0.0)	
Total, *N* = 288 387		
Ethnicity		
White	95 192 (40)	54 554 (18.7)
Mixed	3334 (1.4)	
Asian or Asian British	63 897 (27)	
Black or Black British	38 520 (16)	
Other/Not stated/Unknown	35 799 (15)	
Total, *N* = 236 742		
National IMD decile groups		
(group 1 most deprived, group 4 least deprived)		
Group 1(deciles 1–2)	122 055 (42)	599 (0.2)
Group 2 (deciles 3–4)	84 124 (29)	
Group 3 (deciles 5–6)	42 017 (15)	
Group 4 (deciles 7–10)	42 501 (15)	
Total, *N* = 290 697		
Diabetes type		
Type 1	9880 (4.6)	78 521 (27.0)
Type 2	199 988 (94)	
Not specified	2633 (1.2)	
Other	274 (0.2)	
Total, *N* = 212 775		
Time from diagnosis of diabetes to 1 February 2018		
<5 years	61 220 (32)	99 543 (34.2)
5–9 years	55 901 (29)	
10–19 years	59 887 (31)	
≥20 years	14 745 (7.7)	
Total, *N* = 191 753		

The univariate analysis of Cohort B showed that the following factors were associated with screening attendance: age; sex; ethnicity; diabetes type; time from diagnosis of diabetes; IMD decile; and attendance for first screening appointment within 15 months of registration (Table 3). In terms of age, it was estimated that participants aged 24–29 years were the least likely to attend compared to those aged ≥60 years (OR 0.25, 95% CI 0.22–0.29). The association with age was still present after adjusting for all other factors (adjusted OR 0.31, 95% CI 0.26–0.38). By contrast, sex and type of diabetes were not found to be associated with attendance in the multivariable mixed‐effects logistic regression analysis.

**Table 3:** Mixed‐effects logistic regression analysis with dependent attendance (*N* = 276 335) vs non‐attendance (*N* = 14 960) by the below exploratory factors for all people eligible for screening in Routine Digital Screening (Cohort B).

Exploratory factor	Univariate OR (95% CI)	Multivariable OR (95% CI)
Age at 1 February 2018		
12–17 years	0.73 (0.57–0.95)	1.02 (0.71–1.48)
18–23 years	0.28 (0.24–0.33)	0.33 (0.26–0.42)
24–29 years	0.25 (0.22–0.29)	0.31 (0.26–0.38)
30–35 years	0.27 (0.25–0.30)	0.36 (0.32–0.41)
36–41 years	0.41 (0.38–0.44)	0.50 (0.46–0.55)
42–47 years	0.54 (0.51–0.58)	0.64 (0.59–0.69)
48–53 years	0.65 (0.62–0.69)	0.73 (0.68–0.79)
54–59 years	0.81 (0.76–0.85)	0.87 (0.82–0.93)
≥60 years	Reference	Reference
Sex		
Female	1.05 (1.01–1.08)	0.99 (0.95–1.04)
Male	Reference	Reference
Ethnicity		
White	Reference	Reference
Mixed	0.63 (0.55–0.73)	0.80 (0.67–0.96)
Asian or Asian British	1.19 (1.13–1.25)	1.29 (1.21–1.37)
Black or black British	1.01 (0.96–1.08)	1.03 (0.96–1.11)
Other/not stated/unknown	0.82 (0.75–0.90)	1.04 (0.92–1.17)
National IMD decile groups (group 1 most deprived, group 4 least deprived)		
Group 1 (deciles 1–2)	0.75 (0.69–0.82)	0.73 (0.65–0.82)
Group 2 (deciles 3–4)	0.80 (0.74–0.87)	0.78 (0.70–0.88)
Group 3 (deciles 5–6)	0.91 (0.83–0.99)	0.86 (0.76–0.97)
Group 4 (deciles 7–10)	Reference	Reference
Diabetes type		
Type 1	0.58 (0.53‐0.64)	1.13 (0.99‐1.28)
Type 2	Reference	Reference
Attendance at first screening within 15 months		
No	0.07 (0.07‐0.07)	0.17 (0.16‐0.18)
Yes	Reference	Reference
Time from diagnosis of diabetes to 1 February 2018		
<5 years	Reference	Reference
5–9 years	1.40 (1.33–1.48)	1.21 (1.15–1.29)
10–19 years	1.69 (1.61–1.79)	1.63 (1.54–1.74)
≥20 years	1.58 (1.45–1.73)	1.58 (1.42–1.75)

Abbreviation: IMD, Index of Multiple Deprivation; OR, odds ratio.

Data are ORs of attendance at routine diabetic screening by putative risk factors with 95% CI.

The following factors were associated with the detection of referable retinopathy: age; sex; ethnicity; IMD decile; diabetes type; and time from diagnosis of diabetes. All of these factors were associated with increased odds of requiring further surveillance or referral to the hospital eye service (Table 4). We estimated that people aged 24–29 years at registration were more likely to have referable retinopathy compared to those aged >60 years (adjusted OR 1.85, 95% CI 1.37–2.50). Other risk factors for referable retinopathy included type 1 diabetes, black/minority ethnicity, and living in areas of high socio‐economic deprivation.

**Table 4:** Mixed‐effects logistic regression analysis with dependent referable retinopathy (*N* = 6986) vs no referral (*N* = 215 559) for all people eligible for screening in Routine Digital Screening.

Exploratory factor	Univariate OR (95% CI)	Multivariable OR (95% CI)
Age at 1 February 2018		
12–17 years	0.087 (0.028–0.27)	0.22 (0.07–0.68)
18–23 years	0.69 (0.47–1.00)	1.22 (0.78–1.90)
24–29 years	1.29 (1.02–1.63)	1.85 (1.37–2.50)
30–35 years	1.14 (0.96–1.36)	1.53 (1.19–1.97)
36–41 years	1.20 (1.06–1.35)	2.12 (1.80–2.49)
42–47 years	1.03 (0.94–1.14)	1.82 (1.60–2.07)
48–53 years	1.05 (0.98–1.14)	1.68 (1.52–1.87)
54–59 years	1.01 (0.94–1.08)	1.46 (1.33–1.61)
≥60 years	Reference	Reference
Sex		
Female	0.82 (0.78–0.86)	0.81 (0.76–0.87)
Male	Reference	Reference
Ethnicity		
White	Reference	Reference
Mixed	1.40 (1.09–1.80)	1.24 (0.91–1.69)
Asian or Asian British	1.52 (1.41–1.64)	1.57 (1.43–1.72)
Black or Black British	1.50 (1.38–1.64)	1.65 (1.49–1.83)
Other/not stated/unknown	2.47 (2.28–2.67)	1.78 (1.57–2.02)
National IMD decile groups (group 1 most deprived, group 4 least deprived)		
Group 1 (deciles 1–2)	1.29 (1.18–1.40)	1.42 (1.25–1.62)
Group 2 (deciles 3–4)	1.19 (1.09–1.30)	1.35 (1.19–1.54)
Group 3 (deciles 5–6)	1.10 (1.00–1.22)	1.20 (1.04–1.38)
Group 4 (deciles 7–10)	Reference	Reference
Time from diagnosis of diabetes to 01/02/2018		
<5 years	Reference	Reference
5–9 years	1.59 (1.43–1.77)	1.70 (1.52–1.90)
10–19 years	3.22 (2.93–3.55)	3.74 (3.37–4.15)
≥20 years	7.75 (6.95–8.63)	8.47 (7.48–9.59)
Diabetes type		
Type 1	2.89 (2.62–3.19)	1.74 (1.51–2.02)
Type 2	Reference	Reference

Abbreviation: IMD, Index of Multiple Deprivation; OR, odds ratio.

Odds ratios of referable retinopathy in patients attending routine diabetic screening by putative risk factors.

### 3.2 Visual impairment certification

In 2018/2019, 25 887 new certifications for sight impairment and severe sight impairment in England and Wales were received by the Certifications Office. This corresponds to a rate of 44 per 100 000 population, which was similar to 2009/2010 (Table 5). Over the same period, the rate of vision impairment with diabetic eye disease as the single main reason for certification, fell from 2.4 per 100 000 in 2009/2010 (*n* = 1334) to 1.5 per 100 000 in 2018/2019 (*n* = 904). Similarly, the number of new certifications with a single main cause of diabetic eye disease as a percentage of the total number of certifications fell from 5.5% in 2009/2010 to 3.5% in 2018/2019.

**Table 5:** New certifications for sight impairment and severe sight impairment due to diabetic retinopathy in those persons with known diabetes in England and Wales between 2009 and 2019.

Year	Total	Diabetic eye disease: single main cause	Diabetic eye disease: single main cause, % of total	Diabetic eye disease: single main cause, *n* per 100 000
2009/2010	24 231	1334	*5.5*	2.4
2010/2011	23 926	1261	5.3	3.1
2011/2012	25 079	1329	5.3	2.3
2012/2013	24 009	1194	5.0	2.1
2013/2014	24 213	1066	4.4	1.8
2014/2015	24 260	994	4.1	1.7
2015/2016	24 361	869	3.6	1.5
2016/2017	24 874	903	3.6	1.5
2017/2018	24 299	793	3.3	1.3
2018/2019	25 887	904	3.5	1.5

**Figure 3:**
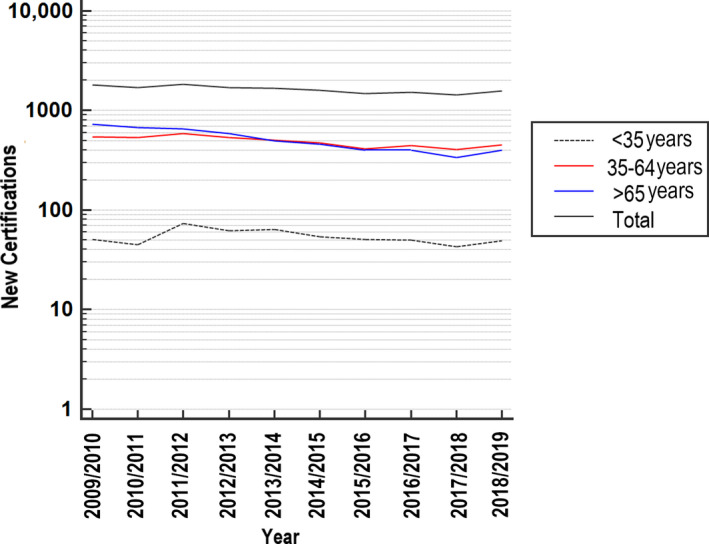
New certifications for sight impairment and severe sight impairment attributable to diabetic retinopathy as the single main cause in England and Wales from 2009 to 2019.

Further analysis of these data based on age showed a consistent downward trend for those aged ≥65 years, and a less pronounced reduction for those aged 35–59 years (Figure 3). By contrast, there was no change in certifiable vision impairment for those aged <35 years over the last 10 years. For this group, 543 young adults were certified as sight‐impaired or severely sight‐impaired in England and Wales over the reporting period.

## 4 DISCUSSION

The primary aim of the present study was to investigate the relationship between age and attendance for diabetic eye screening.

Based on our analysis of Cohort A, the time interval between registration and attendance was significantly longer for the age group 18–34 years, with only 70% meeting the NDESP standard for routine annual screening. More significantly, approximately 20% of young adults were unscreened at the end of the 40‐month reporting period. These results mirror the findings of Scanlon *et al*.,^12^ who similarly found that this group were the least likely to attend promptly for first screening. A longer interval between registration and screening increases the risk of developing sight‐threatening diabetic retinopathy. Scanlon *et al*. reported that the odds of detecting sight‐threatening retinopathy were over four times higher in those whose screening was delayed by 36 months or longer.^12^ In 2017, the NDESP introduced a new quality standard that requires DESPs to report annually the proportion of those who had not attended for screening in the previous 3 years. These data are valuable in identifying high‐risk populations that require targeted interventions to facilitate screening uptake.

The larger sample size of Cohort B provided an opportunity to undertake a more granular analysis of the relationship between age and ongoing screening attendance. Although the overall screening uptake for this cohort was high (~95%), there was considerable variability in uptake between age groups, with young adults being the least likely to attend for screening. Despite participants aged <35 years accounting for <4% of the total cohort, this group had a disproportionately higher rate of non‐attendance (9.3%). Non‐attendance was significantly higher for those aged 24–29 years. The odds of attending a screening event in this group were approximately 70% lower than in the reference group of people aged >60 years, after controlling for other factors. Significantly, young adults were also more likely to present with referable retinopathy. Several published health equity audits over the past two decades have similarly reported on the relative lack of engagement of young people with the DESP.^11^, ^13^, ^18^, ^19^ Regional DESPs report performance against the NDESP standard for uptake of routine digital screening as an aggregated annual figure. Based on the most recent annual report, only two of the regional DESPs in England supplying data failed to meet the ‘acceptable’ performance target of 75% uptake, with 22 meeting the ‘achievable’ 85% target.^9^ However, these figures mask the heterogeneity in screening uptake between demographic groups. Theoretically, robust failsafe systems are in place to identify non‐attenders, auditing post office returns, sending further invitations and executing local initiatives to improve uptake.^20^ Based on the evidence from the present study, there is still a major issue with sub‐optimal attendance in young adults, which highlights the need for policy initiatives and tailored interventions to address these inequities. These could include reporting of attendance by age group and performance indicators for screening uptake being tailored to the risk of developing sight‐threatening retinopathy.

The secondary objective of the present study sought to identify other covariates associated with diabetic eye screening uptake. Data on demographic factors, for example, sex, ethnicity, type and duration of diabetes and social deprivation (IMD deciles), were extracted for each cohort and tested for their association with screening attendance. We identified a consistent socio‐economic gradient in uptake, with the odds of non‐attendance in those living in the most deprived areas (IMD deciles 1–2) being 27% higher than in the least deprived areas (IMD deciles 7–10). Although minority ethnic groups (including both South Asian and African/Afro‐Caribbean people) have a greater likelihood of developing retinopathy than those of white ethnicity,^21^ information on the impact of ethnicity on screening attendance is sparse. This is largely due to the non‐availability of ethnicity data in screening registers.^13^ In our large ethnically diverse sample, self‐reported ethnicity was available for approximately 80% of participants. Using white ethnicity as the reference value, Asian or Asian British people were more likely to attend screening, and those with mixed ethnicity were the least likely to attend. There was no statistical difference in attendance for those of black or black British ethnicity. Significantly, attendance for initial screening within 15 months of registration strongly influenced subsequent screening behaviour; those who failed to attend their initial screening appointment had an 83% lower odds of attending subsequently than those who attended their initial appointment.

Previous studies have retrospectively analysed temporal trends in new certifications of vision impairment in England and Wales attributable to diabetic eye disease.^4^, ^22^, ^23^ In 2014, Liew et al.^22^ reported that, for the first time in at least five decades, diabetic retinopathy/maculopathy was no longer the leading cause of certifiable blindness among working age adults. The present analysis shows that, despite the increasing number of people with diabetes, this downward trend has continued, with 3.5% of new certifications reporting diabetic eye disease as the main cause of vision impairment in 2019 compared to 5.5% in 2009. There are a number of possible explanations for the observed trend, including: the establishment of NDESPs across the UK in 2008, the impact of the Quality and Outcomes Framework on diabetes care that incentivised and resourced general practices to meet key performance indicators in diabetes, and the development of new treatments for diabetic maculopathy. The present study reports, for the first time, the number of new certifications with a single main cause of diabetic eye disease based on age of the population. We identified a marked downward trend over the period 2009 to 2019 for those aged ≥65 years, with a less pronounced reduction for those aged 35–59 years. By contrast, certifications in those aged <35 years over the same period were unchanged. Whilst it is not possible to establish a causal relationship between the development of vision impairment and non‐attendance for diabetic eye screening, given the greater risk of developing sight‐threatening retinopathy in this group, it is likely that failure to attend would delay access to timely and effective treatment for diabetic eye disease.

The main strength of the present study is the use of a large up‐to‐date dataset from a representative multi‐ethnic urban population, with catchment areas that represent the UK’s diversity in terms of vulnerable groups, ethnicity and deprivation. For example, in the Birmingham catchment area there was representation from the first to 10th IMD deciles, with an individual range in rank nationally by Lower Layer Super Output Area from 38 to 32703. Although the dataset was sufficiently large to identify patient‐level factors that influence screening uptake, the lack of geographical variation in included rural populations and the inclusion of different methods of screening, for example, mobile eye screening services, prevented the analysis of these covariates.

For the DESPs in England, the recording of certain information is mandatory, such as the person's name, date of birth, contact details, NHS number, general practitioner, invitation date and screening result. However, the availability of information on ethnicity, type and date of diagnosis of diabetes varies among programmes. From our datasets, which represent three large urban DESPs in England, we found evidence of significant amounts of missing data and variability in data quality among programmes. Important information was often not available. For example, for Cohort B, the type and duration of diabetes were missing for 27% and 34% of records, respectively. Important clinical and lifestyle information is not generally available to the DESP and the ‘General Practice to Diabetic Retinopathy Screening’ system does not currently provide relevant clinical data that would be valuable for failsafe processes in those at higher risk of sight loss. In the future, these data could enable personalized risk‐based screening.^24^

Whilst the NDESP has been successful in ensuring that the vast majority of the eligible population receive retinopathy screening in a timely manner, there is considerable variation in uptake rates among age groups. Young adults with diabetes experience a variety of social and psychological barriers that often lead to poor glycaemic control and non‐engagement with health services.^25^ The present study provides further evidence that a significant minority of young adults are still not engaging with diabetic eye screening services and were more likely to present with sight‐threatening diabetic retinopathy. Those in the age group 18–34 years were the least likely to attend promptly for screening after initial registration and also have the lowest uptake rates at annual screening appointments. This is likely to have major consequences in terms of the risk of developing sight‐threatening diabetic eye disease. Other factors associated with lower uptake rates include living in areas of high socio‐economic deprivation and failure to attend for screening in the first 15 months post‐registration. There is an urgent need for further research to understand patient‐ and system‐level barriers to and enablers of attendance for diabetic eye screening amongst young adults in order to inform policy and development of targeted strategies to increase attendance.

## COMPETING INTERESTS

None declared.
